# New Insights into the Relationship between mIGF-1-Induced Hypertrophy and Ca^2+^ Handling in Differentiated Satellite Cells

**DOI:** 10.1371/journal.pone.0107753

**Published:** 2014-09-17

**Authors:** Simone Guarnieri, Caterina Morabito, Silvia Belia, Laura Barberi, Antonio Musarò, Giorgio Fanò-Illic, Maria A. Mariggiò

**Affiliations:** 1 Department of Neuroscience Imaging and Clinical Sciences, StemTeCh group - CeSI, University G. d'Annunzio of Chieti-Pescara, Chieti, Italy; 2 Interuniversity Institute of Myology (IIM), Italy; 3 Department of Cellular and Environmental Biology, University of Perugia, Perugia, Italy; 4 Institute Pasteur Cenci-Bolognetti, DAHFMO - unit of Histology and Medical Embryology, Sapienza University of Rome, Rome, Italy; 5 Center for Life Nano Science@Sapienza, Istituto Italiano di Tecnologia, Rome, Italy; IRCCS-Policlinico San Donato, Italy

## Abstract

Muscle regeneration involves the activation of satellite cells, is regulated at the genetic and epigenetic levels, and is strongly influenced by gene activation and environmental conditions. The aim of this study was to determine whether the overexpression of mIGF-1 can modify functional features of satellite cells during the differentiation process, particularly in relation to modifications of intracellular Ca^2+^ handling.

Satellite cells were isolated from wild-type and MLC/mIGF-1 transgenic mice. The cells were differentiated *in vitro*, and morphological analyses, intracellular Ca^2+^ measurements, and ionic current recordings were performed.

mIGF-1 overexpression accelerates satellite cell differentiation and promotes myotube hypertrophy. In addition, mIGF-1 overexpression-induced potentiation of myogenesis triggers both quantitative and qualitative changes to the control of intracellular Ca^2+^ handling. In particular, the differentiated MLC/mIGF-1 transgenic myotubes have reduced velocity and amplitude of intracellular Ca^2+^ increases after stimulation with caffeine, KCl and acetylcholine. This appears to be due, at least in part, to changes in the physico-chemical state of the sarcolemma (increased membrane lipid oxidation, increased output currents) and to increased expression of dihydropyridine voltage-operated Ca^2+^ channels. Interestingly, extracellular ATP and GTP evoke intracellular Ca^2+^ mobilization to greater extents in the MLC/mIGF-1 transgenic satellite cells, compared to the wild-type cells.

These data suggest that these MLC/mIGF-1 transgenic satellite cells are more sensitive to trophic stimuli, which can potentiate the effects of mIGF-1 on the myogenic programme.

## Introduction

Insulin-like growth factors (IGFs) represent a system of circulating and/or locally secreted factors that have key roles in cell growth, differentiation and regeneration. Skeletal muscle is a target of the circulating IGFs, and a source of muscle-derived IGF-1 (mIGF-1). IGF-1 also has a critical role in activation, proliferation and differentiation of muscle stem cells (satellite cells), and in tissue repair [1], [2], [3]. During this process, mIGF-1 triggers the activation of *in situ* satellite cells, stimulates the recruitment of circulating stem cells, modulates inflammatory responses, and reduces fibrosis. It has been hypothesized that supplemental mIGF-1 produces a qualitatively different environment for the sustaining of more efficient muscle regeneration and repair [4], [5], [6].

Several processes that are modulated by mIGF-1 have been studied and described in myosin light chain (MLC)/mIGF-1 transgenic mice, an animal model in which the *mIGF-1* gene is regulated by the *MLC* promoter. This restricts the expression of the transgene selectively to skeletal muscle, and predominantly in muscle with a high ratio of fast-twitch fibres [7]. These transgenic mice have an increase in muscle mass (hypertrophy) that is associated with increased force generation. Moreover, the localized up-regulation of *mIGF-1* transgene expression also sustains hypertrophy and regeneration in senescent skeletal muscle [4], [7].

Functional studies have revealed that the hypertrophic phenotype of skeletal muscle in these MLC/mIGF-1 transgenic mice is associated with an increase in muscle strength (tetanic force) and maintained specific force [7], [8], which suggests that increased mIGF-1 levels induce functional hypertrophy and improve functional performance of the skeletal muscle. Recently, using isolated muscle fibres, it was demonstrated that there is lower static stiffness (an index of tissue resistance against deformation in response to an applied force) in these transgenic muscle fibres than in wild-type fibres, and this could be the consequence of an alteration in the expression of the sarcomeric protein titin, and/or modification of the Ca^2+^ sensitivity of the contractile apparatus [9].

In skeletal muscle, the increase in intracellular Ca^2+^ concentration ([Ca^2+^]_i_), that results from motor activation, has a key role in both contractile-activity-dependent and fibre-type-specific gene expression. Mounting evidence has shown that the Ca^2+^/calmodulin-dependent kinases have key roles in the regulation of expression of some of the oxidative enzymes, genesis and activity of the mitochondria, and expression of fibre-type-specific myofibrillar proteins [10]. Alternatively, although the Ca^2+^-activated serine/threonine phosphatase calcineurin is activated by IGF-1 [11], [12] and has well-described functions in the determination of muscle fibre phenotypes [13], this and other Ca^2+^-regulated systems have received little attention as regulators of muscle trophism. Compared to other signalling pathways, the relatively few studies that have examined the role of Ca^2+^ in the regulation of muscle size, have produced discordant results [14], [15], [16].

Therefore, the satellite cells are conditioned by the extracellular muscle microenvironment and are involved in the *in vivo* growth response related to *in situ* mIGF overexpression. For this reason, we used satellite cells from these MLC/mIGF-1 transgenic mice as an experimental model, to determine whether there are any differences in the functional behaviour of these transgenic satellite cells during myogenesis, as compared to those of wild-type mice. The aim of this study was thus to investigate the relationships between increased levels of extracellular mIGF-1 and [Ca^2+^]_i_ handling, as two potential positive factors in the homeostasis of muscle tissue.

## Materials and Methods

### Ethics Statement

The care and use of FVB wild type (WT) and MLC/mIGF-1 transgenic mice (from Musarò Laboratories) strictly followed “The Guiding Principles for the Care and Use of Animals”, in accordance with the principles of the Declaration of Helsinki and with the European Community Council (86/609/CEE) and the Italian Government law on the protection of animals for experimental procedures in research laboratory (92/116). The experimental protocol “Characterization of factors involved in muscle diseases”, which was aimed at the production of the transgenic mice that were used in the present study, was reviewed and approved on 14 February, 2011, by the Institutional Animal Care and Use Committee of the Unit of Histology and Medical Embryology (‘La Sapienza’ University of Rome, Rome, Italy). The animals (3 months old) were sacrificed under sodium pentobarbital anaesthesia and all efforts were made to minimize suffering.

### Chemicals and materials

Unless otherwise indicated, cell culture media, sera and antibiotics were from Life Technologies Italia (Monza, Italy), cell culture plastic-ware was from Becton Dickinson Falcon (Sacco Srl, Cadorago, Italy), and the reagents and standards were from Sigma-Aldrich (Milan, Italy).

### Satellite cell isolation

The animals (3 months old) were sacrificed under sodium pentobarbital anaesthesia. The bulk of hind-limb muscles were harvested under sterile conditions, and then manually shredded and digested for 30 min at 37°C with 1 mg ml^−1^ collagenase/dispase (Roche Diagnostics, Mannheim, Germany) in phosphate-buffered saline (PBS). After centrifugation (150× *g* for 5 min), the pellet was digested for 15 min at 37°C with 0.1 mg ml^−1^ collagenase type II (Sigma-Aldrich) in PBS. The enzymatic reaction was blocked by adding cell growth medium (high-glucose Dulbecco's modified Eagle's medium supplemented with 20% horse serum, 3% chick embryonic extract, 4 mM L-glutamine, 100 IU ml^−1^ penicillin, and 100 µg ml^−1^ streptomycin), then the suspension was filtered (40 micron cell strainer filter; Falcon), and then centrifuged at 200× *g* for 15 min. The pellet of cells was re-suspended in growth medium, and the cells were pre-plated twice for 1 h, to remove the fibroblasts. The satellite cells were then plated on collagen-coated dishes and grown in growth medium [17]. To differentiate the cells into myotubes, after 5 days of culture, the medium of the satellite cell cultures was changed to the differentiation medium (Dulbecco's modified Eagle's medium supplemented with 5% horse serum, 4 mM L-glutamine, and 100 IU ml^−1^ penicillin, and 100 µg ml^−1^ streptomycin) for 10 days [18].

The level of successful differentiation of the satellite cells into myotubes was determined using immuno-staining protocols, with an antibody against myosin heavy chain (anti-MyHC; MF20 Hybridoma Bank, Iowa, USA), and an antibody against MyoD (Santa Cruz Biotechnology Inc), accompanied by Hoechst staining of the nuclei. To assess the level of myogenic differentiation, the number of Hoechst-stained nuclei located within the sarcomeric myosin-positive cells, and of MyoD-positive cells was determined and expressed as:

- the myogenic differentiation (differentiation %), calculated as the number of Hoechst-stained nuclei located within sarcomeric myosin-positive cells, and expressed as a percentage of total analysed myogenic cells, positive to MyoD;
- the fusion index, quantified as the percentage of Hoechst-stained nuclei located within multinucleated cells (at least 2 nuclei, thus having undergone fusion), positive to sarcomeric myosin, and calculated on the total analysed MyoD-positive nuclei;
- myonuclear accretion, expressed as average number of nuclei per myotube, and relative percentage of mature myotubes (containing at least 5 nuclei) [19].

The morphometric analyses were performed calculating the size and length of MyHC-positive myotubes, using Image J (National Institutes of Health, Frederick, MD) [19].

To better define the percentage of different populations present in culture we performed cytofluorimetric analysis, following protocols described by Carosio et al [20]. We performed additional analysis 2 days after plating, by immuno-fluorescence analysis for the expression of MyoD (Santa Cruz Biotechnology Inc), and α-smooth muscle actin (anti-α-SMA, Sigma-Aldrich), a marker of myofibroblasts. We revealed that our muscle-derived primary cultures contain an average of 40% of MyoD-positive cells and an average 20% of α-smooth muscle actin-positive cells.

### RNA extraction and real time PCR analysis

Total RNA extraction was performed using TriRiagent (Sigma-Aldrich) and was reverse-transcribed using the QuantiTec Reverse Transcription kit (QIAGEN srl, Milan, Italy). Relative quantitative PCR was performed on ABI PRISM 7500 SDS (Applied Biosystems, USA), using premade 6-carboxyfluorescein (FAM)-labelled TaqMan assays for HPRT1 (Mm00446968_m1), MyoD1 (Mm00440387_m1), Myogenin (Mm00446194_m1), Pax-7 (Mm00834079_m1), Myh-2 (Mm00454991_m1), IGF-1 (Mm00710307_m1) and MRF4 (Mm00435126_m1) (Applied Biosystem). Relative quantitative real time PCR (RT-PCR) sample value was normalized for the expression of HPRT1 mRNA. The relative level for each gene was calculated using the 2-ddCt method and reported as mean fold change in gene expression.

### Measurement of intracellular Ca^2^ in single cells

The [Ca^2+^]_i_ was measured using the dye Fura-2-acetoxymethyl ester (Fura-2/AM; Molecular Probes, Life Technologies Italia, Monza, Italy), with an inverted microscope (Olympus Italia S.r.l., Segrate, Milan) connected to a high-speed wavelength switcher (Polychrome II; Till Photonics, Germany) equipped with a 75-W stabilized xenon lamp (Ushio, Japan) and a cooled charge-coupled device (CCD) camera (C6790 model; Hamamatsu Photonics, Hamamatsu, Japan). The wild-type and MLC/mIGF-1 transgenic satellite cells and myotubes plated on collagen-coated special-optics 96-well plates (Corning-Costar, distributed by Sigma-Aldrich) were incubated with 5 µM Fura-2/AM for 30 min at 37°C in normal external solution (140 mM NaCl, 2.8 mM KCl, 2 mM CaCl_2_, 2 mM MgCl_2_, 10 mM glucose, 10 mM N-2-hydroxyethilpiperazione-N′-2-ethanesulfonic acid (HEPES)-NaOH, pH 7.3) supplemented with 1% (w/v) bovine serum albumin. Fura-2/AM loaded cells were sequentially and repetitively excited at 340 nm and 380 nm, the fluorescence images were acquired with a CCD camera and stored on an interfaced computer. The acquisition time was one image ratio (i.e., 340/380 signal) per second. The image-ratio calculations were carried out pixel-by-pixel on a pair of corresponding 340 nm and 380 nm image files. The temporal plots (mean fluorescence signal in a selected cell area) were calculated from the image ratios. The [Ca^2+^]_i_ in a single cell field was recorded according to the [Ca^2+^]_i_ calibration plot of the 340/380 ratio, as calculated using the Calcium Calibration kits for video imaging (Molecular Probes) [21].

### Dihydropyridine receptor Ca^2+^ channel binding assay

This binding assay was performed with membranes purified from the wild-type and MLC/mIGF-1 mature myotubes sonicated in sodium phosphate buffer (20 mM NaPO_4_, pH 7.0) supplemented with protease inhibitors according to a procedure described by Fulle et al. [22]. The dihydropyridine receptor (DHPR) concentrations were determined using the radioligand [^3^H]-PN200-110. The membrane proteins (40 µg) were incubated in a final volume of 250 µl binding buffer (200 mM KCl, 10 mM HEPES, 100 mM CaCl_2_, 0.1 mM diisopropylfluorophosphate, and 1 µg µl^−1^ leupeptin, pH 7.4) in the presence of 1 nM [^3^H]-PN200-110 for 1 h at room temperature. The samples were then filtered through Whatman GF/C filters and rapidly washed with six volumes of ice-cold washing buffer (200 mM KCl, 10 mM HEPES, pH 7.4). The radioactivity associated with the filters (membrane-bound [^3^H]-PN200-110) was then determined by liquid scintillation counting (LS 6500 Multi-Purpose Counter, Beckman Coulter, Fullerton, CA, USA), and is expressed as pmol mg^−1^ protein. The non-specific [^3^H]-PN200-110 binding to the membranes was determined in the presence of 10 µM unlabelled nifedipine, and this value was subtracted from each experimental point [23].

### Ryanodine receptor Ca^2+^ channel binding assay

This ryanodine receptor (RyR) binding assay was performed on whole homogenates obtained from sonicated wild-type and MLC/mIGF-1 transgenic mature myotubes (25 µg protein assay^−1^), as indicated for the DHPR binding assay above. The samples were incubated in 250 µl binding buffer in the presence of 5 nM [^3^H]-ryanodine for 120 min at 37°C. The samples were then filtered through Whatman GF/C filters and rapidly washed with six volumes of ice-cold washing buffer. The radioactivity associated with the filters (membrane-bound [^3^H]-ryanodine) was then determined by liquid scintillation counting (LS 6500 Multi-Purpose Scintillation Counter, Beckman Coulter), and is expressed as pmol mg^−1^ protein. The non-specific [^3^H]-ryanodine binding was determined in the presence of 100 µM unlabelled ryanodine, and this value was subtracted from each experimental point [23].

### Ca^2+^/Mg^2+^ ATPase assay

The assay for the determination of the Ca^2+^/Mg^2+^ ATPase activity was carried out on whole homogenates obtained from sonicated myotubes (as for the radiolabelled binding assays). Each sample contained 25 µg protein incubated in 1 ml containing 2.5 mM ATP, 100 µM CaCl_2_, 60 µM K-EGTA, 10 mM KCl, 5 mM MgCl_2_, 300 mM sucrose, 10 mM HEPES, pH 7.4, for 30 min at room temperature. The reactions were terminated with the addition of 1 ml 12.5% trichloroacetic acid, and the precipitate was removed by centrifugation at 5000× *g* for 10 min. The released phosphates were estimated from 1 ml of the cleared supernatant, according to the method described previously [23]. The specific Ca^2+^/Mg^2+^ ATPase activity was calculated as µg released inorganic phosphate (Pi) min^−1^ ml^−1^ mg^−1^ protein.

### Western blotting

The wild-type and MLC/mIGF-1 transgenic myotubes were washed with pre-cooled phosphate-buffered saline and sonicated in 20 mM sodium phosphate buffer (pH 7.0) containing 100 µg ml^−1^ phenylmethylsulfonyl fluoride, 10 µg ml^−1^ leupeptin, 5 µg ml^−1^ pepstatin A, and 10 µg ml^−1^ benzamidine. The sonicated cells were centrifuged at 1000× *g* for 10 min at 4°C, the supernatants were collected, and the protein concentrations were determined using the Bio-Rad protein assay (Bio-Rad Laboratories). Samples containing 50 µg protein were resuspended in Laemmli buffer (8% [w/v] sodium dodecyl sulphate [SDS], 10% [v/v] glycerol, 5% [v/v] β-mercaptoethanol, 25 mM Tris-HCl, pH 6.5, and 0.003% [w/v] bromophenol blue), boiled for 5 min, and separated by SDS–PAGE on 6% (w/v) gels for DHPR and RyR1 protein detection, and 8% (w/v) gels for that of Ca^2+^-ATPase. The proteins were electroblotted onto a hydrophobic polyvinylidene difluoride membrane (Immobilon-P membrane; Millipore, Bedford, MA, USA). These membranes were blocked in TBS-T (Tris-buffered saline with 0.1% [v/v] Tween 20) containing 5% (w/v) fat-free milk, and then incubated individually with mouse monoclonal antibodies against DHPR (anti-DHPR alpha 1 antibody; Abcam Ltd, Cambridge, UK; dilution, 1∶1000), against RyR1 (Clone 34 C; Sigma-Aldrich; dilution, 1∶5000), and against Ca^2+^-ATPase (anti-SERCA1 antibody; Abcam Ltd; dilution, 1∶3000). The membranes were then incubated with horseradish-peroxidase-conjugated anti-mouse IgG (dilution, 1∶10000), which was detected by chemiluminescence (ECL Plus; GE–Amersham, Little Chalfont, UK). An anti-actin antibody (Sigma-Aldrich; dilution, 1∶1000) was used as a loading control. All of the antibodies were diluted in TBS-T.

### Malondialdehyde measurements and membrane fluidity assay

To define the oxidative status of the cell membrane lipids, malondialdehyde levels were determined. Malondialdehyde forms an adduct with thiobarbituric acid that can be measured spectrophotometrically. The measurement of such thiobarbituric acid reactive substances is a well-established method for screening and monitoring of lipid peroxidation. These lipid peroxidation measurements on the sarcolemma and sarcoplasmic reticulum membranes isolated from the wild-type and MLC/mIGF-1 transgenic skeletal muscle, were performed using the OXItek ‘TBARS’ Assay kits (ZeptoMetrix Corp., Buffalo, NY, USA), as described by the manufacturer. The sarcolemma and sarcoplasmic reticulum membrane fractions were prepared as previously described [24].

Membrane fluidity was assayed using the MarkerGene Membrane Fluidity Kit (Marker Gene Technologies, Inc., Eugene, OR, USA), that contains a lipophilic pyrene probe that undergo excimer formation upon spatial interaction, and consequently shifting its emission spectrum. This property makes it a useful tool to monitor on live cells, changes in membrane fluidity or viscosity. The protocol was optimized using different cell number (1000, 3000 or 6000 cells/well), 1–50 µM fluorescent lipid reagent concentrations, dye-incubation times (15–30 min) and temperatures (20–45°C). The results, shown here, come from experiments performed on wild-type and MLC/mIGF-1 transgenic satellite cells plated (3000 cells/well) on collagen-coated special-optics 96-well plates (Corning-Costar). After 0, 3 and 7 days in differentiation medium, cells were incubated with 10 µM fluorescent lipid reagent (Membrane Fluidity Kit) for 20 min at 25°C also in accordance with the manufacturer instructions. At the end, the probe was removed, cells washed twice with PBS and the residual fluorescence read at 360 nm excitation wavelength, and 470 or 400 nm emission wavelength, using a microplate reader (SpectraMAX Gemini XS, Molecular Devices, Toronto, ON, Canada). Membrane fluidity was expressed as the ratio of fluorescent intensities at 470 and 400 nm emission wavelength (excimer/monomer).

### Electrophysiological measurements

In voltage-clamp experiments, the total currents were recorded using an extracellular solution containing 130 mM NaCl, 3 mM KCl, 1 mM CaCl_2_, 2 mM MgCl_2_, 10 mM glucose, and 10 mM HEPES-HCl, pH 7.4). The patch pipette was filled with 130 mM KCl, 2 mM MgCl_2_, 10 mM glucose, 5 mM EGTA and 10 mM HEPES-NaOH (pH 7.4). The electrophysiological recordings were carried out at room temperature using the whole-cell configuration of the patch-clamp technique [25], [26]. Stimulation, acquisition, and data analysis were performed using the pCLAMP 9.0 and Clampfit software (Axon Instruments, Burlingame, CA, USA). The cells were clamped to a holding potential of −90 mV and the currents recorded from −100 mV to +65 mV, in increments of 5 mV. The components of the leak and capacitive currents were cancelled using the P/N method. The patch pipettes had a resistance of 3 MΩ to 6 MΩ, and to reduce their capacitance, their tips were coated with Sylgard. A sampling interval of 25 µs point^−1^ was used, and the currents were filtered at 5 kHz.

### Statistical analysis

All of the quantitative data are expressed as means ± standard error of the mean (SEM). Differences between groups were analysed using Students' t-tests, with the Prism 4 software (GraphPad Software, San Diego, CA, USA).

## Results

### Satellite cells from wild-type and MLC/mIGF-1 transgenic mice

**Figure 1:**
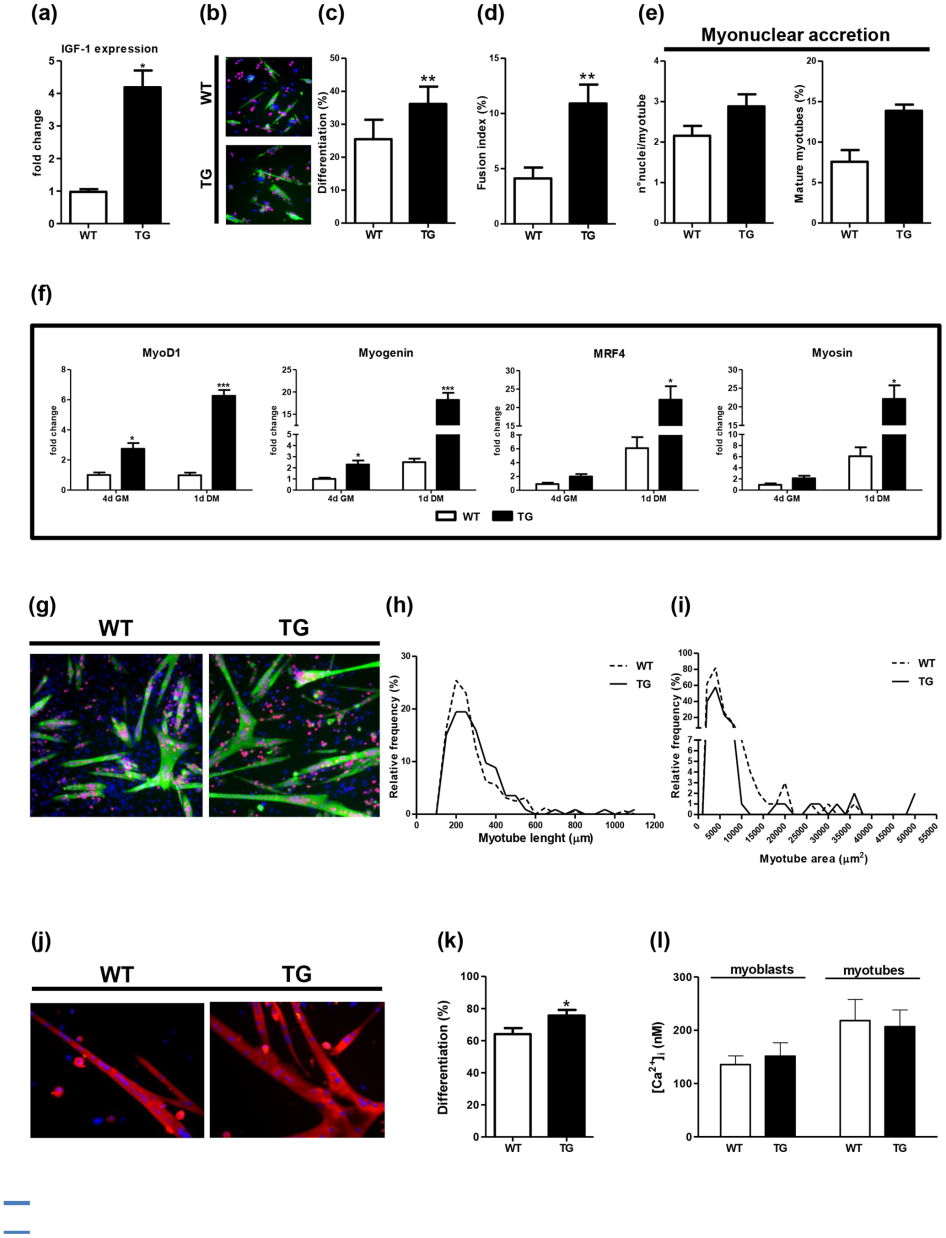
Post-mitotic expression of mIGF-1 accelerates satellite cell differentiation. (a) RT-PCR analysis of endogenous mIGF-1 expression in 5-day differentiated satellite cells isolated from wild-type (WT) and MLC/mIGF-1 transgenic (TG) mice. (b–f) Analyses on WT and TG mouse-derived cells after 4 days in growth medium: representative images from WT (b-top panel) and TG (b-bottom panel) mouse-derived cells immuno-stained for myosin (green fluorescence) and MyoD (red fluorescence), accompanied by Hoechst-stained nuclei (blue fluorescence); differentiation index (differentiation %) (c); fusion index (d); myonuclear accretion expressed as average number of nuclei per myotube (e-left panel), and relative percentage of mature myotubes (e-right panel); RT-PCR analyses of MyoD1, Myogenin, MRF4 and Myosin (f). (g–i) Analyses on WT and TG mouse-derived cells after 1 day in differentiation medium: representative images from WT (g-left panel) and TG (g-right panel) mouse-derived cells immuno-stained for myosin (green fluorescence) and MyoD (red fluorescence), accompanied by Hoechst-stained nuclei (blue fluorescence); morphometric analyses expressed as relative frequency of myotubes' length (h) and area (i). (j and k) Analyses on WT and TG mouse-derived cells after 5 days in differentiation medium: representative images from WT (j-left panel) and TG (j-right panel) mouse-derived cells immuno-stained for myosin (red fluorescence), accompanied by Hoechst-stained nuclei (blue fluorescence), and differentiation index (differentiation %) (k). (l) Basal intracellular Ca^2+^ concentrations ([Ca^2+^]_i_) measured in undifferentiated (myoblasts) and differentiated (myotubes) satellite cells derived from WT and TG mice muscle. Data in panels a, c–f, k are means ±SEM from 3 independent experiments, data in panel l are means ±SEM from 165 wild-type myoblasts, 101 wild-type myotubes, 98 MLC/mIGF-1 transgenic myoblasts, and 67 MLC/mIGF-1 transgenic myotubes, as assayed from three independent experiments for each type. * p<0.05, ** p<0.01 and *** p<0.001 *versus* wild-type.

Satellite cells were isolated from the hind-limb muscles of wild-type and MLC/mIGF-1 transgenic mice. As expected, and previously published [7], [27], the transgene mIGF-1 was not expressed in quiescent and proliferating satellite cells, whereas it was expressed in post-mitotic myoblasts and accumulated in differentiated myotubes (data not shown). We also analysed the expression of endogenous mIGF-1 in both wild-type and MLC/mIGF-1 transgenic mouse-derived myotubes (5 days in differentiation medium). RT-PCR analysis revealed a significant increased expression of mIGF-1 in differentiated cells isolated from the transgenic animals compared to wild-type differentiated cells (Fig. 1a).

We then analysed the effects of mIGF-1 on myogenic program, performing a time course experiment. We observed that mIGF-1 expression accelerates the differentiation of satellite cells and promotes a precocious mature phenotype even at early stage of the myogenic program (4 days in growth medium) (Fig. 1b–f). In particular we observed that MLC/mIGF-1 transgenic mouse-derived cells, compared to wild-type cell cultures, showed: i) a higher myogenic differentiation (differentiation %, calculated as the number of Hoechst-stained nuclei located within sarcomeric myosin-positive cells, and expressed as a percentage of total analysed myogenic cells, positive to MyoD) (Fig. 1c), ii) a higher fusion index (quantified as the percentage of Hoechst-stained nuclei located within multinucleated cells, positive to sarcomeric myosin, and calculated on the total analysed MyoD-positive nuclei (Fig. 1d), and iii) a myonuclear accretion (expressed as average number of nuclei per myotubes, figure 1e-left panel, and relative percentage of mature myotubes, figure 1e-right panel).

The accelerated process of myogenic differentiation, induced by mIGF-1 expression, was also supported by the significant up-regulation of relevant markers involved in myogenic differentiation, such as MyoD1, Myogenin, MRF4 and myosin, in MLC/mIGF-1 transgenic mouse-derived cells compared to wild-type cell cultures (Fig. 1f).

Morphometric analyses, performed at 1 day in differentiation medium, revealed that the MLC/mIGF-1 transgenic mouse-derived myotubes appeared longer and with a bigger area compared to the wild-type mouse–derived myotubes (Fig. 1g–i).

The effect of mIGF-1 overexpression on the myogenic process was also confirmed at 5 days in differentiation medium: at this time MLC/mIGF1 transgenic mouse-derived cultures showed an increased myogenic differentiation and larger myotubes compared to the wild-type mouse–derived cultures (Fig. 1j and k).

These data correlate with the muscle phenotype of the transgenic mice [7] and support the maturation effects of mIGF-1 on myogenic cells [11].

### Intracellular Ca^2+^

Calcium has key roles in many intracellular processes. Here, [Ca^2+^]_i_ was monitored in these isolated satellite cells, using fluorescence video-imaging techniques. Figure 1k shows that during differentiation, [Ca^2+^]_i_ increased by about 50% in the wild-type differentiated cells and 30% in the MLC/mIGF-1 transgenic differentiated cells. This increase was significant (p = 0.0194, non-parametric unpaired test between the combined myoblast and myotube populations), although there was no significant difference in the [Ca^2+^]_i_ rise between the wild-type and MLC/mIGF-1 transgenic models (Fig. 1l).

**Figure 2:**
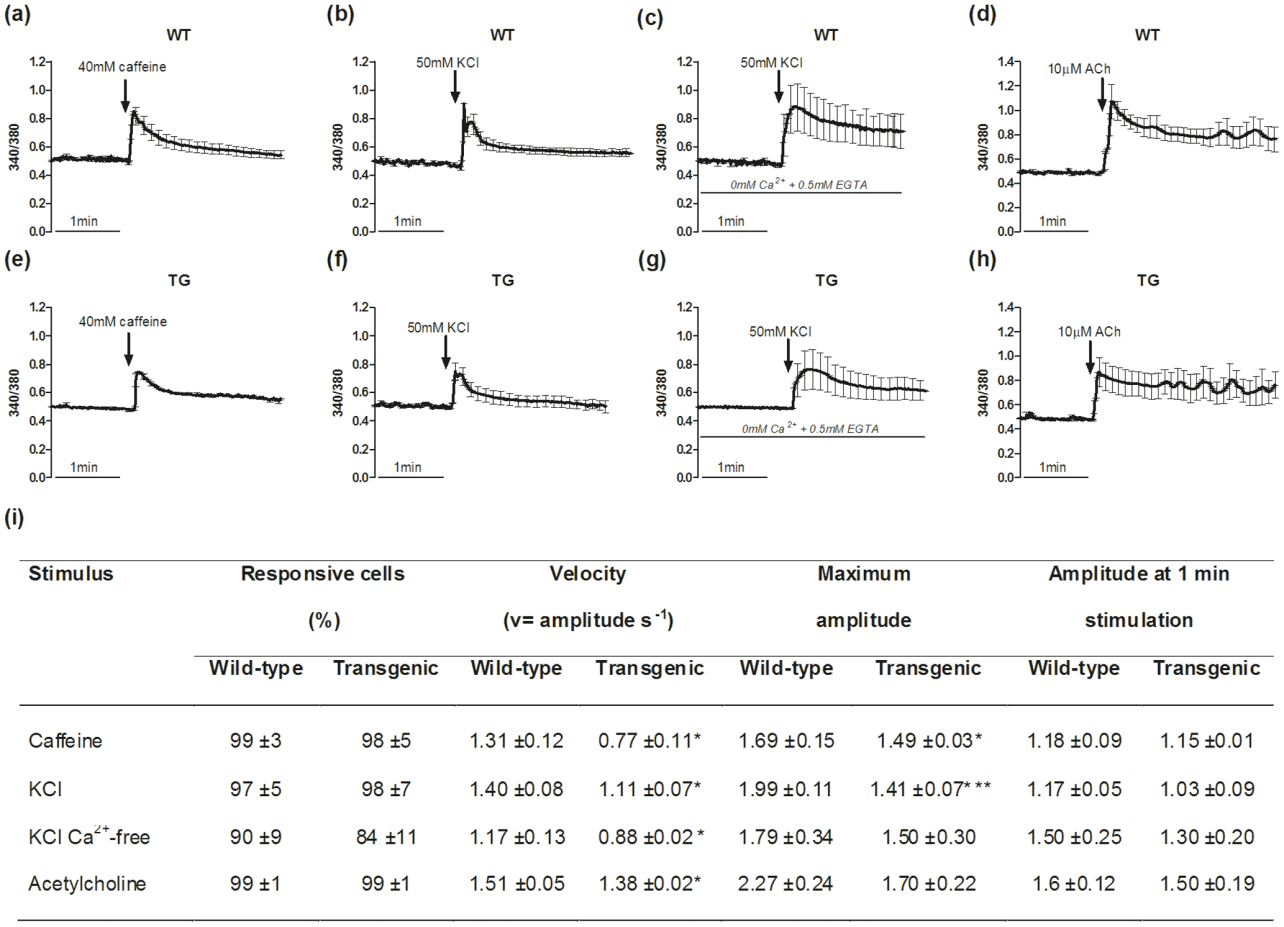
Intracellular Ca^2+^ variations in myotubes. (a–h) Representative intracellular Ca^2+^ variations in the wild-type (WT) and MLC/mIGF-1 transgenic (TG) myotubes expressed as fluorescence ratios (340/380). The time courses were recorded during addition of caffeine (a, e), KCl with (b, f) and without (c, g) extracellular Ca^2+^, and acetylcholine (ACh; d, h). (i) Quantification of intracellular Ca^2+^ response parameters determined for the wild-type and MLC/mIGF-1 transgenic mature myotubes. They include: the percentage of cells responsive to each stimulus, the Velocity to reach the peak of Ca^2+^ increase, calculated as amplitude to time to peak (v = amplitude s^−1^); Maximum amplitude calculated as the ratio of the F_340/380_ at the peak to the basal F_340/380_; Amplitude at 1 min stimulation calculated as the ratio of the F_340/380_ at 1 min from application of stimulus to the basal F_340/380_. * p<0.05; *** p<0.001.

The *in vitro* differentiation process yielded myotubes that were functional, as these showed increases in intracellular Ca^2+^ levels in response to the RyR Ca^2+^ channel agonist (40 mM caffeine; Fig. 2a, e) and to a depolarizing agent (50 mM KCl; Fig. 2b, f), also in the absence of extracellular calcium (Fig. 2c, g). No significant responses were seen in the presence of caffeine or KCl in the proliferating myoblasts (data not shown).

The quantitative analysis of the ion kinetics (Fig. 2i) shows that there was a slower intracellular Ca^2+^ level increase evoked by caffeine in the MLC/mIGF-1 transgenic myotubes compared to the wild-type myotubes (v = 0.77±0.11 vs. 1.31±0.12, respectively; p<0.05). Similarly, in the presence of extracellular Ca^2+^, KCl induced a significantly slower [Ca^2+^]_i_ increase in the MLC/mIGF-1 transgenic myotubes compared to the wild-type myotubes (v = 1.11±0.07 vs. 1.40±0.08; p<0.05); this was also seen with KCl in Ca^2+^-free medium (v = 0.88±0.02 vs 1.17±0.13; p<0.05). In the absence of extracellular Ca^2+^, only the myotubes with complete maturation of their excitation-contraction coupling showed intracellular Ca^2+^ level increases, like those illustrated for myotubes in Figure 2c, g.

The initial peak of the caffeine-induced and KCl-induced intracellular Ca^2+^ level increases (as the immediate response) was significantly lower in the myotubes derived from the MLC/mIGF-1 transgenic mice compared to that measured in the wild-type cultures (Fig. 2i). However, this difference was not seen in the response at 1 min after stimulus administration, when the levels of Ca^2+^ in the two models were similar (Fig. 2). In order to test the behaviour of the DHPR in the two cell phenotypes, also 1 µM Bay-K8644, a specific channel agonist, was used. 1 µM Bay-K8644 induced an intracellular Ca^2+^ increase in a quite similar manner to that observed with KCl. Kinetics' analysis of [Ca^2+^]i variations evoked by Bay-K8644 showed a significant decrease of the maximum amplitude in MLC/mIGF-1 transgenic myotubes (2.01±0.12, p<0.05) compared to that observed in the wild-type myotubes (2.50±0.17). Moreover, also the amplitude at 1 min stimulation was very similar to that induced by KCl, showing no significant differences between MLC/mIGF-1 transgenic myotubes (1.28±0.06) and wild-type myotubes (1.14±0.05).

The responsiveness to acetylcholine mimicked that for caffeine and KCl, with a decreased response velocity in MLC/mIGF-1 transgenic myotubes compared to wild-type myotubes (v = 1.38±0.02 vs. 1.51±0.05; p<0.05; Fig. 2d, h, i).

**Figure 3:**
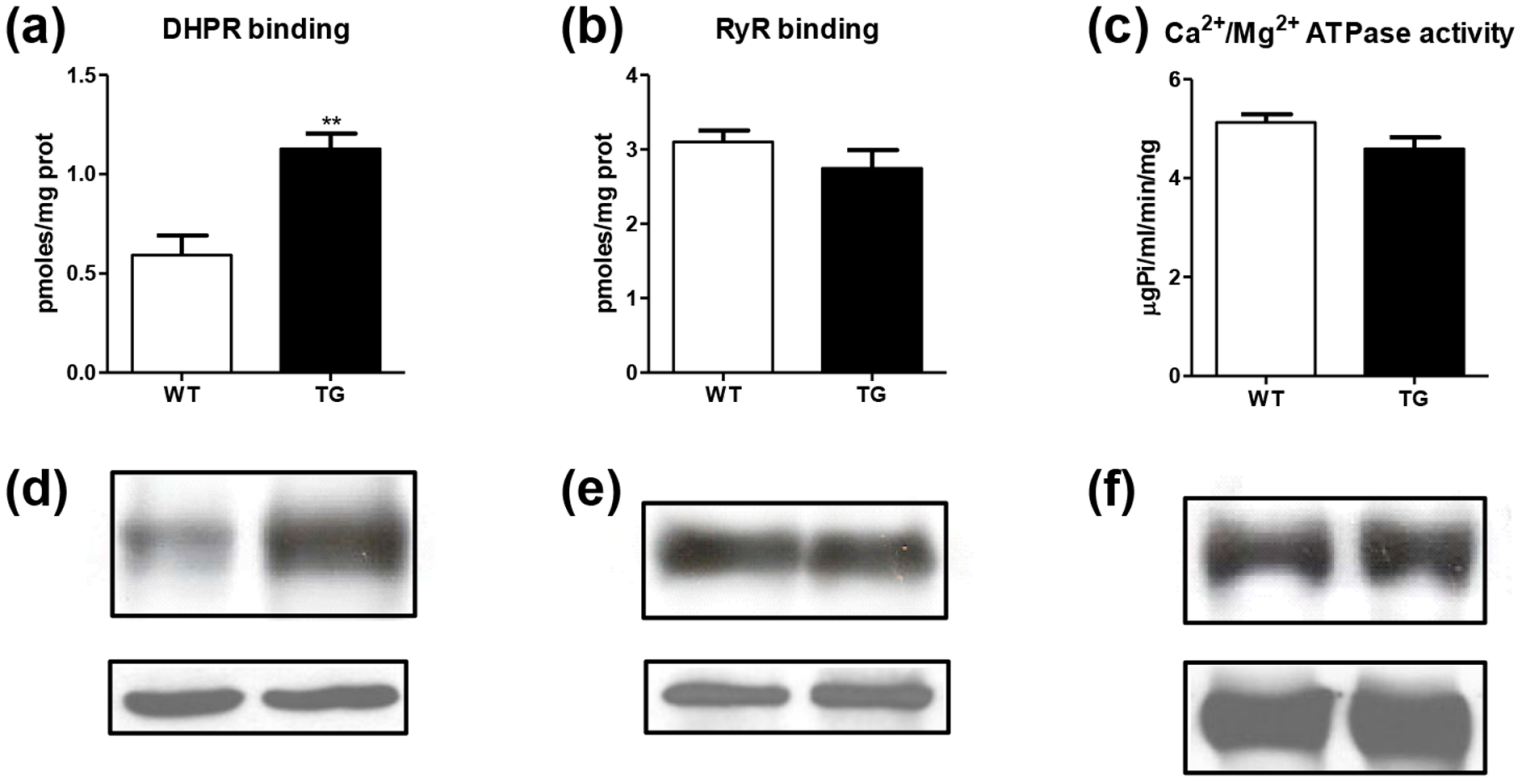
Analysis of the major proteins involved in intracellular Ca^2+^ homeostasis. Membranes from wild-type (WT) and MLC/mIGF-1 transgenic (TG) myotubes were analysed for binding of radiolabelled [^3^H]-PN200-110 to DHPR Ca^2+^ channels (a), and radiolabelled [^3^H]-ryanodine to RyR Ca^2+^ channels (b), and for Ca^2+^/Mg^2+^ ATPase activity (c). Data are means ±SEM of three independent experiments, ** p<0.01. Representative immunoblots of the expression levels are also shown, for DHPR (d), RyR (e), and the Ca^2+^/Mg^2+^ ATPase protein (f); actin was used as loading control.

These data can be related to the different capacities of these systems that control the Ca^2+^ handling. In addition, binding experiments and immunoblotting of membrane preparations from the wild-type and MLC/mIGF-1 transgenic myotubes revealed that the MLC/mIGF-1 transgenic samples showed significantly higher levels of the DHPR voltage-operated Ca^2+^ channels, compared to wild-type (Fig. 3a and d). Conversely, the RyR channel levels (Fig. 3b and e) and the Ca^2+^/Mg^2+^ pump activities and levels (Fig. 3c and f) were not significantly different.

**Figure 4:**
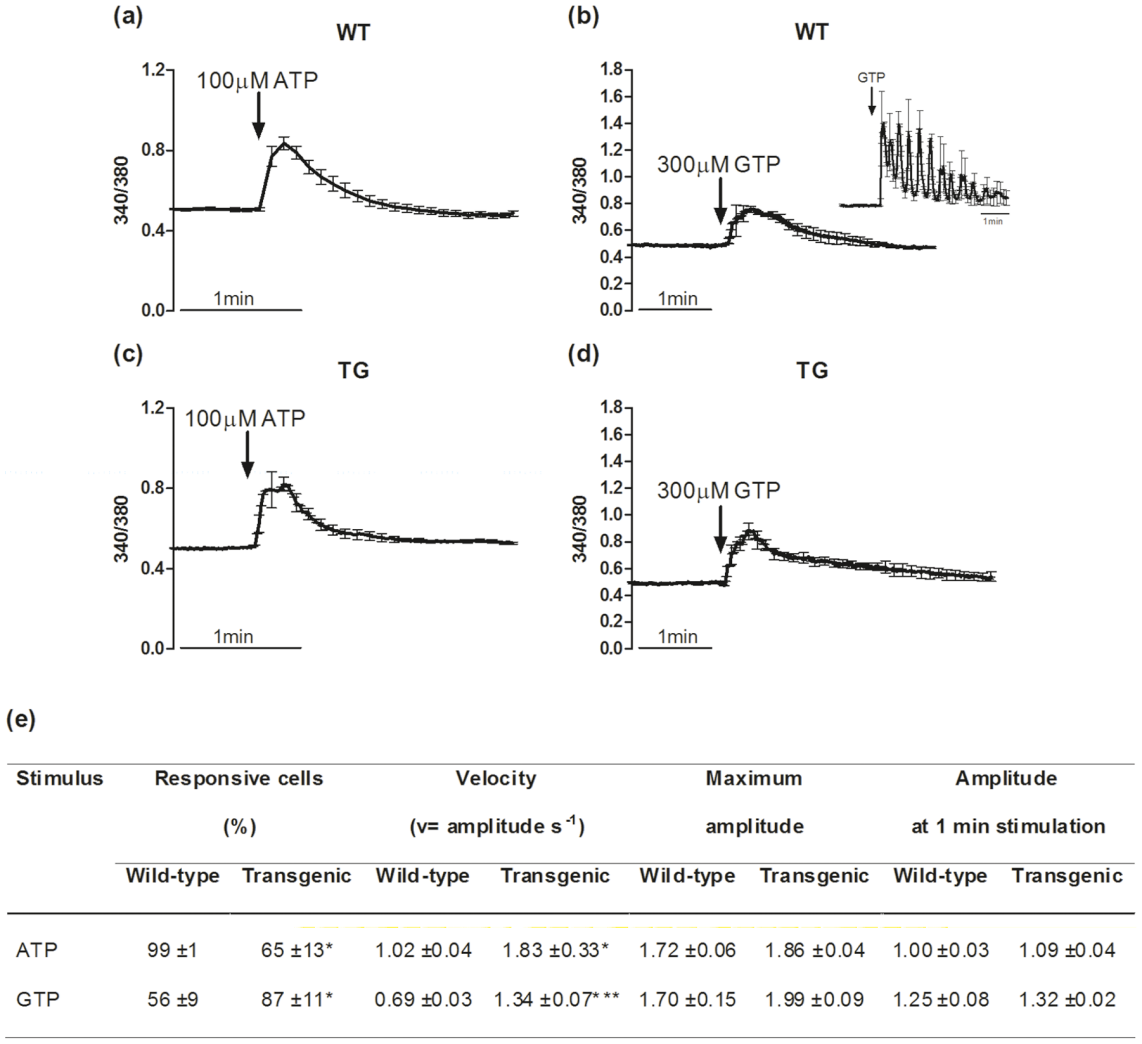
Purine-induced intracellular Ca^2+^ rises in myoblasts. (a–d) Representative intracellular Ca^2+^ variations in the wild-type (WT) and MLC/mIGF-1 transgenic (TG) myoblasts, expressed as fluorescence ratios (340/380). The time courses were recorded during the addition of ATP (a, c) and GTP (b, d). (e) Quantification of intracellular Ca^2+^ response parameters determined for the wild-type and MLC/mIGF-1 transgenic myoblasts. They include: the percentage of cells responsive to each stimulus, the Velocity to reach the peak of Ca^2+^ increase, calculated as amplitude to time to peak (v = amplitude s^−1^); Maximum amplitude calculated as the ratio of the F_340/380_ at the peak to the basal F_340/380_; Amplitude at 1 min stimulation calculated as the ratio of the F_340/380_ at 1 min from application of stimulus to the basal F_340/380_. * p<0.05; *** p<0.001.

In satellite cells, as well as in other *in vitro* muscle cell models, intracellular Ca^2+^ level increases are triggered also by myogenic stimuli, such as purines, which are known for their trophic actions that are mediated by changes in intracellular Ca^2+^ levels [28], [29], [30]. Under these conditions, the wild-type and MLC/mIGF-1 transgenic myoblasts showed different behaviours for these stimulus-evoked intracellular Ca^2+^ level increases (Fig. 4). Both ATP and GTP affected the Ca^2+^ handling parameters (amplitude, response velocity) in the wild-type and MLC/mIGF-1 transgenic cells. In particular, both 100 µM ATP and 300 µM GTP induced significantly more rapid intracellular Ca^2+^ level increases in MLC/mIGF-1 transgenic myoblasts compared to those evoked in wild-type myoblasts (Fig. 4).

Of note, the wild-type and MLC/mIGF-1 transgenic myoblast populations showed different behaviours to these purine treatments: the MLC/mIGF-1 transgenic cells were significantly less sensitive to ATP (65%±13% responsive cells) compared to the wild-type myoblasts (99%±1%); conversely, the MLC/mIGF-1 transgenic myoblasts were significantly more responsive to GTP (87%±11%) compared to the wild-type myoblasts (56%±9%) (Fig. 4e). In addition, in about half of the responsive wild-type myoblasts, GTP induced intracellular Ca^2+^ level increases that showed oscillations that lasted for 2–5 min, and that occurred with a frequency of about one every 30 s (Fig. 4b, inset). This phenomenon was not seen in the MLC/mIGF-1 transgenic myoblasts.

**Figure 5:**
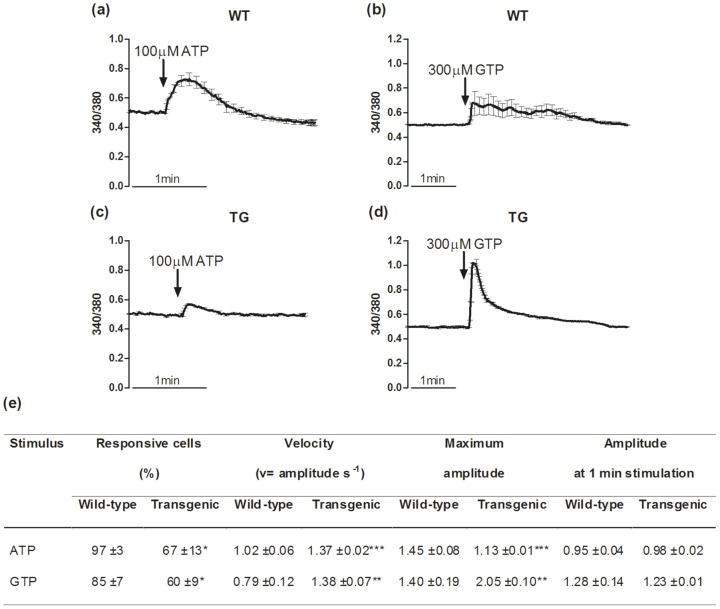
Purine-induced intracellular Ca^2+^ rises in myotubes. (a–d) Representative intracellular Ca^2+^ variations in the wild-type (WT) and MLC/mIGF-1 transgenic (TG) myotubes, expressed as fluorescence ratios (340/380). The time courses were recorded during the addition of ATP (a, c) and GTP (b, d). (e) Quantification of intracellular Ca^2+^ response parameters determined for the wild-type and MLC/mIGF-1 transgenic myotubes. They include: the percentage of cells responsive to each stimulus, the Velocity to reach the peak of Ca^2+^ increase, calculated as amplitude to time to peak (v = amplitude s^−1^); Maximum amplitude calculated as the ratio of the F_340/380_ at the peak to the basal F_340/380_; Amplitude at 1 min stimulation calculated as the ratio of the F_340/380_ at 1 min from application of stimulus to the basal F_340/380_. * p<0.05; ** p<0.01; *** p<0.001.

When the same experimental approach was used with the myotubes, differences were observed in the responsiveness and the intracellular Ca^2+^ level increase parameters (amplitude, response velocity) in the presence of these purines. In particular, the intracellular Ca^2+^ level increases stimulated by both ATP and GTP showed significantly lower cell responsiveness in the MLC/mIGF-1 transgenic myotubes (67%±13%, 60%±9%, respectively) in comparison to the wild-type myotubes (97%±3%, 85%±7%, respectively) (Fig. 5e). The amplitude of the ATP-induced intracellular Ca^2+^ level increases was significantly lower for the transgenic MLC/mIGF-1 myotubes in comparison to the wild-type myotubes (1.13±0.01 vs. 1.45±0.08; p<0.001), while the GTP-responsive cells in the MLC/mIGF-1 transgenic myotubes showed a significantly higher amplitude of the intracellular Ca^2+^ level increase with respect to the wild-type myotubes (2.05±0.10 vs. 1.40±0.19; p<0.01) (Fig. 5e). Under these conditions, these differences in the amplitudes of the intracellular Ca^2+^ level increases were no longer apparent in the response at 1 min after purine administration (Fig. 5). Concerning the velocity of the intracellular Ca^2+^ level increase, as for the myoblasts, this was greater in the MLC/mIGF-1 transgenic myotubes with respect to the wild-type myotubes, for both ATP and GTP stimulation (Fig. 5).

### Electrical properties

To support the functional background of the satellite cells, the membrane electrical properties and the inward and outward currents were also measured in myoblasts and myotubes from the wild-type and MLC/mIGF-1 transgenic samples. These analyses were performed using the patch-clamp technique in the whole-cell configuration [25].

**Figure 6:**
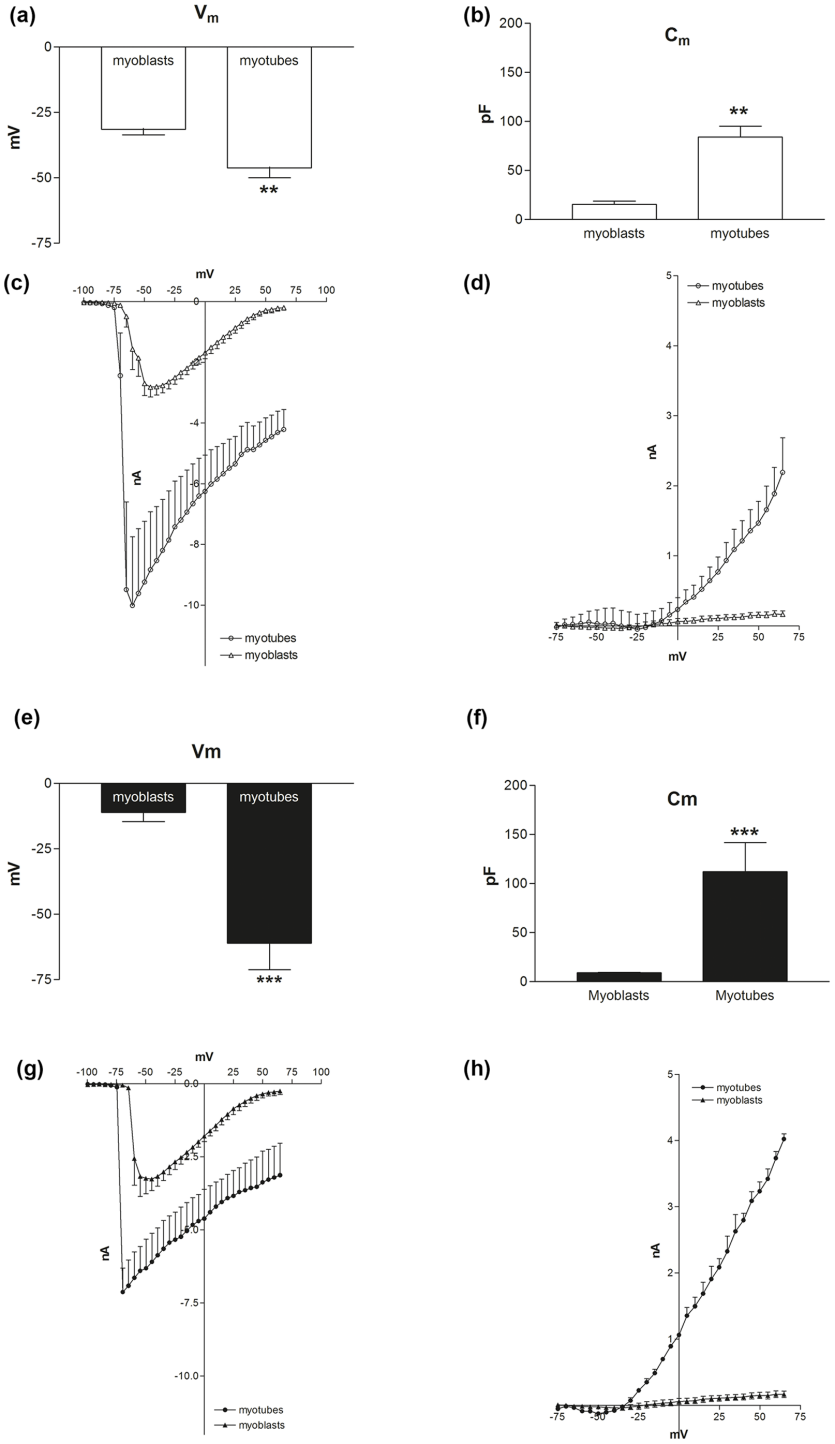
Electrical properties and ionic currents in wild-type (a–d) and MLC/mIGF-1 transgenic (e–h) myoblasts and myotubes. Recordings of the resting potential, Vm (a, e), cell capacitance, Cm (b, f), inward current component (c, g), and outward current component (d, h). Data are means ±SEM, from 15 wild-type myoblasts, 11 wild-type myotubes, 13 MLC/mIGF-1 transgenic myoblasts, and 17 MLC/mIGF-1 transgenic myotubes, from three independent experiments for each cellular phenotype.

After the satellite cell differentiation, the resting potential (Vm) was significantly lower in the myotubes with respect to the myoblasts, for both the wild-type and MLC/mIGF-1 transgenic samples (Fig. 6a, e). Although this did not reach significance, the MLC/mIGF-1 transgenic myotubes showed a lower Vm with respect to the wild-type myotubes (Fig. 6a, e). The membrane capacitance (Cm) was significantly increased in myotubes with respect to their myoblast precursors, in both wild-type and MLC/mIGF-1 transgenic samples, although without significant differences between the wild-type and the MLC/mIGF-1 transgenic samples (Fig. 6b, f). Also, the current-voltage relationship (I/V) calculated from the inward and outward components of the total current measurements revealed increased inward currents in both the wild-type and MLC/mIGF-1 transgenic myotubes, with respect to their corresponding myoblasts, again without any significant difference between the wild-type and the MLC/mIGF-1 transgenic samples (Fig. 6c, g). The only difference between the wild-type and MLC/mIGF-1 transgenic samples revealed by this experimental approach, was for the outward currents, which were significantly more pronounced in the MLC/mIGF-1 transgenic myotubes with respect to the wild-type myotubes (p<0.05) (Fig. 6d, h).

### Oxidative status and fluidity of the cell membranes

The functional activity of myotubes depends not only on the differentiative status, but also on the environmental conditions. The oxidative balance in skeletal muscle is one of the main features that is representative of its metabolic activity as a whole, and this also conditions the satellite cell activity. For these reasons, the oxidative status of cell membranes was assayed in mature skeletal muscle biopsies from the wild-type and the MLC/mIGF-1 transgenic mice.

**Figure 7:**
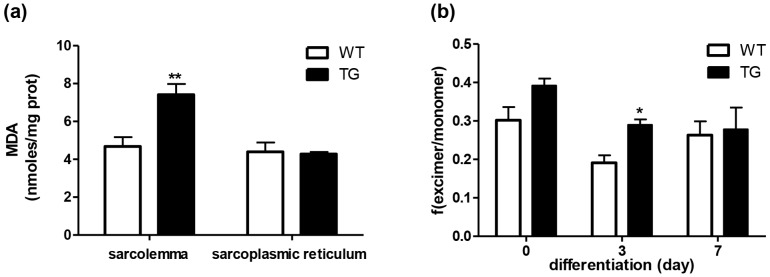
Quantification of the oxidative status and fluidity of the cell membrane lipids. (a) Malondialdehyde levels were determined in the sarcolemma and sarcoplasmic reticulum membrane fractions from skeletal muscle biopsies of the wild-type (WT) and MLC/mIGF-1 transgenic (TG) mice. (b) Membrane fluidity of WT and TG satellite cells, measured at 0, 3 and 7 days in differentiation medium, and expressed as the ratio of fluorescence intensities at 470 and 400 nm emission wavelength (excimer/monomer). Data are means ±SEM of three independent experiments. * p<0.05, ** p<0.01 *versus* wild-type.

Figure 7a shows that there were significantly higher levels of malondialdehyde (as a measure of the oxidative status of cell membrane lipids) in the sarcolemmal fractions from the MLC/mIGF-1 transgenic samples compared to the wild-type samples (p<0.01), while there was no difference for the sarcoplasmic reticulum membrane fractions (Fig. 7a).

The oxidative status of lipids can affect the membrane biophysical properties, and for this reason, membranes' fluidity was assayed on live MLC/mIGF-1 and wild-type differentiating satellite cells at 0, 3 and 7 days in differentiation medium, using a fluorescent probe. The results showed some differences in membranes' fluidity between MLC/mIGF-1 transgenic and wild-type satellite cells. The differences were significant at 3 days of the differentiation period (Fig. 7b).

## Discussion

It has been previously demonstrated that *mIGF-1* gene expression induces local skeletal muscle hypertrophy, which improves muscle mass and strength by activating calcineurin-mediated Ca^2+^ signalling and GATA-2/NF-ATc1 [11]. Similarly, treatment of C2C12 skeletal muscle cells with the recombinant IGF-1 protein elicits similar effects, with the promotion of hypertrophy via the calcineurin pathway [12].

Interestingly, Zheng and co-workers proposed a model in which CaM kinase and calcineurin are involved in IGF-1-induced DHPRalpha expression in C2C12 cells. IGF-1 receptor activation triggers CaM kinase and calcineurin activity to regulate the balance of phosphorylated/dephosphorylated CREB, that in turn regulates DHPRalpha transcription [31].

More recently, Li and co-workers [32] provided evidence that a transmembrane protein located in the sarcoplasmic reticulum, named STIM1, acts as a Ca^2+^ sensor and activates Ca^2+^ entry by the Orai channels. STIM1 has a fundamental role in postnatal skeletal muscle growth in relation to the calcineurin/NF-AT signalling pathways, through the regulation of the levels of Ca^2+^ in the sarcoplasm and sarcoplasmic reticulum cisternae. Based on this mechanism, it was proposed a model whereby STIM1-mediated store-operated Ca^2+^ entry regulates the Ca^2+^ signalling required for cellular processes that are necessary for muscle growth and differentiation, even though the excitation-contraction coupling remains unperturbed [32].

Many evidences demonstrate the deep relationship between growth factor-induced intracellular Ca^2+^-dependent signalling and muscle hypertrophy, and that support the working hypotheses developed in the present study. The data presented here relating to some of the systems involved in intracellular Ca^2+^ homeostasis in myotubes (DHPR, RyR, Ca^2+^ pumps), show that the differences in the behaviour observed in MLC/mIGF-1 transgenic myotubes in comparison to the wild-type myotubes arise from the different kinetics of the Ca^2+^-controlling system, rather than from any further specific component.

In the myotubes derived from the MLC/mIGF-1 transgenic mice, we observed increased capacity of the T-tubule–associated DHPR channels, together with increased outward potassium currents, with respect to the myotubes derived from the wild-type mice. *Vice versa*, in the MLC/mIGF-1 transgenic myotubes, only a small, and not significantly different, decrease in the functional capacity of the sarcoplasmic reticulum was observed, in terms of the RyR channels and Ca-pumps.

Overall, these data suggest that in the myotubes derived from the MLC/mIGF-1 transgenic cultures, Ca^2+^ mobilization was slower under all of the conditions tested here (caffeine, depolarization via KCl, in the presence or absence of external Ca^2+^, acetyl choline), even if after longer times (≥1 min), the differences between the MLC/mIGF-1 transgenic and wild-type cultures were significantly reduced.

These data indicated that in the MLC/mIGF-1 transgenic myotubes, there might be different sarcolemmal organization, as both the increased DHPR functionality and the increased output K^+^ currents are a possible consequence of external membrane modifications that do not involves the sarcoplasmic reticulum structures. The data derived from the oxidative status of the sarcolemma appear to confirm this hypothesis. These show that in the membranes derived from mature myotubes, the lipid oxidation is greater in the MLC/mIGF-1 transgenic samples compared to wild-type ones, which suggests modification of the membrane fluidity [33]. Measurements on live differentiating MLC/mIGF-1 transgenic satellite cells showed a significant increase of membranes' fluidity in respect to the wild-type cells. This evidence supports the hypothesis that there is a different membranes' organization in MLC/mIGF-1 transgenic satellite cells, even if a deeper analysis is required to define the mechanism linking membrane oxidation and its biophysical features.

The electrical events are also very important during myoblast differentiation. It has been reported that during differentiation of human muscle satellite cells, the resting potential is hyperpolarized, due to the sequential expression and activation of ionic channels. Such hyperpolarization, which is mainly due to the activation of ether-a-go-go and inward rectifier K^+^ channels, leads to increased Ca^2+^ influx, which promotes myoblasts fusion and consequently multinucleated myotube formation [34]. This condition appears to be reproduced also in our experimental models, in which the myotubes derived from MLC/mIGF-1 transgenic cell cultures show a higher hyperpolarized state and outward currents (mainly K^+^ currents) in comparison to the wild-type myotubes, which supports the hypothesis that these cells are more prone to differentiate.

In skeletal muscle, there is another Ca^2+^-activating system that can influence myogenesis: the extracellular pool of micromolar concentrations of purines, such as ATP and GTP [35]. Muscle cells can release purines as a consequence of microlesions, or more extensive lesions (as well as during physical exercise), or of the lowering of the pH [36]. Therefore purines can act on the same skeletal muscle cells with autocrine and paracrine mechanisms, with the activation of P2 and/or P2-like receptors [37], [38]. In the skeletal muscle C2C12 cell line, the activation of ATP and GTP receptors leads to intracellular Ca^2+^ level increases that are due to the release of Ca^2+^ from intracellular inositol-1,4,5-trisphosphate-sensitive stores [29]. In differentiating C2C12 myoblasts, as well as in other cell models, extracellular GTP activates a metabotropic cascade that leads to transient intracellular Ca^2+^ mobilization, the consequent activation of the intermediate Ca^2+^-activated K^+^ channels (IK_Ca_), and hyperpolarization of the plasma membrane [39], [40]. At the same time, Ca^2+^ released from internal stores induces a proliferative boost, and increases the number of cells that express the myosin heavy chain proteins and at least three genes involved in myogenesis: *Pp3ca*, *Gsk3b* and *Pax7* [40], [41]. Taking into account that due to the position (below the basal lamina) of the satellite cells they are directly influenced by the activity of the muscle fibres, it is possible that any modification of the fibre ‘niche’, including released factors and purines, can affect the level of activation of satellite cells.

Our data show that at physiological external concentrations, ATP and GTP induce a more rapid Ca^2+^ mobilization in muscle-derived cells derived from the mIGF-1 transgenic mice, as compared to the wild-type cells. During repetitive stimulation of skeletal muscle, extracellular ATP levels raise, which activates purinergic receptors, and thus increases Ca^2+^ influx, and enhances the contractile force, a response that is known as potentiation [42]. In cultured myotubes, this ATP-induced potentiation might be represented by the greater velocity of the Ca^2+^ rise rather than a higher Ca^2+^ level, which might be due to a more controlled *in vitro* extracellular environment with respect to what happens *in vivo*. This evidence might explain the potentiation induced in the MLC/mIGF-1 transgenic mice by ATP and GTP, which induced higher and more rapid Ca^2+^ responses. In addition, considering that GTP can also potentiate muscle activity [43], [44], we can hypothesize that the repetitive contractions induced in the mIGF-1 transgenic mice might evoke a ‘potentiation effect’ higher than that obtained in the wild-type muscles. Del Prete and colleagues [8] showed that in MLC/mIGF-1 transgenic mice, the fast-twitch fibres of the extensor digitorum longus had increased absolute tetanic force and absolute maximum power, with respect to those of the wild-type extensor digitorum longus. This finding is not in contrast with the possibility that the purines secreted during contraction (from many fibres simultaneously) can reach levels that can induce the potentiation of the whole muscle.

The data in the present study show that about 50% of the wild-type myoblasts are responsive to 300 µM GTP, which increases to about 90% in the MLC/mIGF-1 transgenic myoblasts. This response appears to provide evidence of the better myogenic properties of the MLC/mIGF-1 transgenic cells, considering that GTP acts via two specific binding sites with different affinities, and in particular, that only the activation of the low-affinity GTP-binding site favours the fusion and myogenesis processes [29], [40].

Of note, in the wild-type cells, GTP stimulated Ca^2+^ oscillations, which was not seen in the MLC/mIGF-1 transgenic myoblasts. It has been hypothesized that the molecular mechanisms responsible for intracellular Ca^2+^ oscillations depends on the biphasic (release/uptake) regulation of the inositol 1,4,5-trisphosphate receptor by the cytosolic Ca^2+^. Generally, high frequency cytosolic Ca^2+^ oscillations regulate fast responses, such as synaptic transmission and secretion, whereas lower frequency oscillations regulate slower processes, such as gene transcription [45]. In addition, physiological stimulation can drive gene expression by triggering local Ca^2+^ influxes, and not global intracellular Ca^2+^ oscillations [46]. Thus, in cells that are conditioned by a continuous and persistent mIGF-1 action, as in those from the MLC/mIGF-1 transgenic mice, the response to additive differentiative factors, such as GTP, might be modified, such as the early intracellular Ca^2+^ level increase. Under the experimental conditions of the present study, the GTP-induced intracellular Ca^2+^ oscillations, observed in wild-type myoblasts, are replaced by the more rapid intracellular Ca^2+^ level increase in the MLC/mIGF-1 transgenic cells.

In conclusion, the data provided by the present study indicate that the functional properties of these MLC/mIGF-1 transgenic satellite cells are mostly different from those of the wild-type cells. In particular, this mIGF-1 overexpression leads to changes in the control of intracellular Ca^2+^ handling, these are both quantitative (velocity, amplitude) and qualitative (absence of the GTP-induced intracellular Ca^2+^ oscillations). This is due, at least in part, to changes in the physico-chemical state of the sarcolemma (increased oxidation of membrane lipids, increased output currents) and to increased expression of DHPR voltage-operated Ca^2+^ channels.

Of particular interest, ATP and GTP evoked intracellular Ca^2+^ mobilization to a greater extent in the MLC/mIGF-1 transgenic cells, with respect to wild-type cells, and this might explain both the potentiation and activation of myogenesis. Although further investigations are necessary to define whether these changes are directly involved in the mechanism(s) underlying the hypertrophic status of the transgenic muscle, it can be hypothesized that the sensitivity of these MLC/mIGF-1 transgenic cells towards non-canonical myogenic factors, such as the purines, appears to be a specific mechanism involved in the effects induced *in vivo* by the overexpression of mIGF-1.

## References

[1] Adams GR (2002) Invited Review: Autocrine/paracrine IGF-I and skeletal muscle adaptation. J Appl Physiol (1985) 93: 1159–1167. 12183514 10.1152/japplphysiol.01264.2001

[2] Edwall D, Schalling M, Jennische E, Norstedt G (1989) Induction of insulin-like growth factor I messenger ribonucleic acid during regeneration of rat skeletal muscle. Endocrinology 124: 820–825. 2912704 10.1210/endo-124-2-820

[3] Philippou A, Maridaki M, Halapas A, Koutsilieris M (2007) The role of the insulin-like growth factor 1 (IGF-1) in skeletal muscle physiology. In Vivo 21: 45–54. 17354613

[4] Musarò A, Giacinti C, Borsellino G, Dobrowolny G, Pelosi L, et al (2004) Stem cell-mediated muscle regeneration is enhanced by local isoform of insulin-like growth factor 1. Proc Natl Acad Sci U S A 101: 1206–1210. 14745025 10.1073/pnas.0303792101 PMC337031

[5] Pelosi L, Giacinti C, Nardis C, Borsellino G, Rizzuto E, et al (2007) Local expression of IGF-1 accelerates muscle regeneration by rapidly modulating inflammatory cytokines and chemokines. FASEB J 21: 1393–1402. 17264161 10.1096/fj.06-7690com

[6] Scicchitano BM, Rizzuto E, Musarò A (2009) Counteracting muscle wasting in aging and neuromuscular diseases: the critical role of IGF-1. Aging (Albany NY) 1: 451–457. 20157530 10.18632/aging.100050 PMC2806025

[7] Musarò A, McCullagh K, Paul A, Houghton L, Dobrowolny G, et al (2001) Localized IGF-1 transgene expression sustains hypertrophy and regeneration in senescent skeletal muscle. Nat Genet 27: 195–200. 11175789 10.1038/84839

[8] Del Prete Z, Musarò A, Rizzuto E (2008) Measuring mechanical properties, including isotonic fatigue, of fast and slow MLC/mIGF-1 transgenic skeletal muscle. Ann Biomed Eng 36: 1281–1290. 18415017 10.1007/s10439-008-9496-x

[9] Colombini B, Benelli G, Nocella M, Musarò A, Cecchi G, et al (2009) Mechanical properties of intact single fibres from wild-type and MLC/mIGF-1 transgenic mouse muscle. J Muscle Res Cell Motil 30: 199–207. 19731048 10.1007/s10974-009-9187-8

[10] Chin ER (2005) Role of Ca^2+^/calmodulin-dependent kinases in skeletal muscle plasticity. J Appl Physiol (1985) 99: 414–423. 16020436 10.1152/japplphysiol.00015.2005

[11] Musarò A, McCullagh KJ, Naya FJ, Olson EN, Rosenthal N (1999) IGF-1 induces skeletal myocyte hypertrophy through calcineurin in association with GATA-2 and NF-ATc1. Nature 400: 581–585. 10448862 10.1038/23060

[12] Semsarian C, Wu MJ, Ju YK, Marciniec T, Yeoh T, et al (1999) Skeletal muscle hypertrophy is mediated by a Ca^2+^-dependent calcineurin signalling pathway. Nature 400: 576–581. 10448861 10.1038/23054

[13] Schiaffino S (2010) Fibre types in skeletal muscle: a personal account. Acta Physiol (Oxf) 199: 451–463. 20353491 10.1111/j.1748-1716.2010.02130.x

[14] Hudson MB, Price SR (2013) Calcineurin: a poorly understood regulator of muscle mass. Int J Biochem Cell Biol 45: 2173–2178. 23838168 10.1016/j.biocel.2013.06.029 PMC3947871

[15] Wang ZM, Messi ML, Delbono O (2002) Sustained overexpression of IGF-1 prevents age-dependent decrease in charge movement and intracellular Ca^2+^ in mouse skeletal muscle. Biophys J 82: 1338–1344. 11867450 10.1016/S0006-3495(02)75489-1 PMC1301936

[16] Wang ZM, Messi ML, Renganathan M, Delbono O (1999) Insulin-like growth factor-1 enhances rat skeletal muscle charge movement and L-type Ca^2+^ channel gene expression. J Physiol 516 Pt 2: 331–341. 10087334 10.1111/j.1469-7793.1999.0331v.x PMC2269266

[17] Musarò A, Barberi L (2010) Isolation and culture of mouse satellite cells. Methods Mol Biol 633: 101–111. 20204623 10.1007/978-1-59745-019-5_8

[18] Guarnieri S, Morabito C, Paolini C, Boncompagni S, Pilla R, et al (2013) Growth associated protein 43 is expressed in skeletal muscle fibers and is localized in proximity of mitochondria and calcium release units. PLoS One 8: e53267. 23308181 10.1371/journal.pone.0053267 PMC3538766

[19] Markworth JF, Cameron-Smith D (2013) Arachidonic acid supplementation enhances in vitro skeletal muscle cell growth via a COX-2-dependent pathway. Am J Physiol Cell Physiol 304: C56–67. 23076795 10.1152/ajpcell.00038.2012

[20] Carosio S, Barberi L, Rizzuto E, Nicoletti C, Del Prete Z, et al (2013) Generation of eX vivo-vascularized Muscle Engineered Tissue (X-MET). Sci Rep 3: 1420. 23478253 10.1038/srep01420 PMC3594753

[21] Mariggiò MA, Falone S, Morabito C, Guarnieri S, Mirabilio A, et al (2010) Peripheral blood lymphocytes: a model for monitoring physiological adaptation to high altitude. High Alt Med Biol 11: 333–342. 21190502 10.1089/ham.2009.1097

[22] Fulle S, Belia S, Vecchiet J, Morabito C, Vecchiet L, et al (2003) Modification of the functional capacity of sarcoplasmic reticulum membranes in patients suffering from chronic fatigue syndrome. Neuromuscul Disord 13: 479–484. 12899875 10.1016/s0960-8966(03)00042-7

[23] Belia S, Santilli F, Beccafico S, De Feudis L, Morabito C, et al (2009) Oxidative-induced membrane damage in diabetes lymphocytes: effects on intracellular Ca^2+^ homeostasis. Free Radic Res 43: 138–148. 19115119 10.1080/10715760802629588

[24] Hemmings SJ (2001) New methods for the isolation of skeletal muscle sarcolemma and sarcoplasmic reticulum allowing a comparison between the mammalian and amphibian beta(2)-adrenergic receptors and calcium pumps. Cell Biochem Funct 19: 133–141. 11335938 10.1002/cbf.909

[25] Guarnieri S, Pilla R, Morabito C, Sacchetti S, Mancinelli R, et al (2009) Extracellular guanosine and GTP promote expression of differentiation markers and induce S-phase cell-cycle arrest in human SH-SY5Y neuroblastoma cells. Int J Dev Neurosci 27: 135–147. 19111604 10.1016/j.ijdevneu.2008.11.007

[26] Hamill OP, Marty A, Neher E, Sakmann B, Sigworth FJ (1981) Improved patch-clamp techniques for high-resolution current recording from cells and cell-free membrane patches. Pflugers Arch 391: 85–100. 6270629 10.1007/BF00656997

[27] Musarò A, Rosenthal N (1999) Maturation of the myogenic program is induced by postmitotic expression of insulin-like growth factor I. Mol Cell Biol 19: 3115–3124. 10082578 10.1128/mcb.19.4.3115 PMC84105

[28] Lorenzon P, Bandi E, de Guarrini F, Pietrangelo T, Schafer R, et al (2004) Ageing affects the differentiation potential of human myoblasts. Exp Gerontol 39: 1545–1554. 15501025 10.1016/j.exger.2004.07.008

[29] Pietrangelo T, Mariggiò MA, Lorenzon P, Fulle S, Protasi F, et al (2002) Characterization of specific GTP binding sites in C2C12 mouse skeletal muscle cells. J Muscle Res Cell Motil 23: 107–118. 12416717 10.1023/a:1020288117082

[30] Ryten M, Dunn PM, Neary JT, Burnstock G (2002) ATP regulates the differentiation of mammalian skeletal muscle by activation of a P2X5 receptor on satellite cells. J Cell Biol 158: 345–355. 12135987 10.1083/jcb.200202025 PMC2173112

[31] Zheng Z, Wang ZM, Delbono O (2004) Ca(2+) calmodulin kinase and calcineurin mediate IGF-1-induced skeletal muscle dihydropyridine receptor alpha(1S) transcription. J Membr Biol 197: 101–112. 15014912 10.1007/s00232-003-0645-8

[32] Li T, Finch EA, Graham V, Zhang ZS, Ding JD, et al (2012) STIM1-Ca^2+^ signaling is required for the hypertrophic growth of skeletal muscle in mice. Mol Cell Biol 32: 3009–3017. 22645307 10.1128/MCB.06599-11 PMC3434510

[33] Fulle S, Mecocci P, Fanò G, Vecchiet I, Vecchini A, et al (2000) Specific oxidative alterations in vastus lateralis muscle of patients with the diagnosis of chronic fatigue syndrome. Free Radic Biol Med 29: 1252–1259. 11118815 10.1016/s0891-5849(00)00419-6

[34] Bernheim L, Bader CR (2002) Human myoblast differentiation: Ca^2+^ channels are activated by K^+^ channels. News Physiol Sci 17: 22–26. 11821532

[35] Burnstock G, Arnett TR, Orriss IR (2013) Purinergic signalling in the musculoskeletal system. Purinergic Signal 9: 541–572. 23943493 10.1007/s11302-013-9381-4 PMC3889393

[36] Tu J, Lu L, Cai W, Ballard HJ (2012) cAMP/protein kinase A activates cystic fibrosis transmembrane conductance regulator for ATP release from rat skeletal muscle during low pH or contractions. PLoS One 7: e50157. 23226244 10.1371/journal.pone.0050157 PMC3511434

[37] Borno A, Ploug T, Bune LT, Rosenmeier JB, Thaning P (2012) Purinergic receptors expressed in human skeletal muscle fibres. Purinergic Signal 8: 255–264. 22052557 10.1007/s11302-011-9279-y PMC3350594

[38] Pietrangelo T, Guarnieri S, Fulle S, Fanò G, Mariggiò MA (2006) Signal transduction events induced by extracellular guanosine 5′ triphosphate in excitable cells. Purinergic Signal 2: 633–636. 18404466 10.1007/s11302-006-9021-3 PMC2096655

[39] Guarnieri S, Fano G, Rathbone MP, Mariggiò MA (2004) Cooperation in signal transduction of extracellular guanosine 5′ triphosphate and nerve growth factor in neuronal differentiation of PC12 cells. Neuroscience 128: 697–712. 15464278 10.1016/j.neuroscience.2004.06.073

[40] Pietrangelo T, Fioretti B, Mancinelli R, Catacuzzeno L, Franciolini F, et al (2006) Extracellular guanosine-5′-triphosphate modulates myogenesis via intermediate Ca^2+^-activated K^+^ currents in C2C12 mouse cells. J Physiol 572: 721–733. 16455689 10.1113/jphysiol.2005.102194 PMC1780011

[41] Mancinelli R, Pietrangelo T, Burnstock G, Fanò G, Fulle S (2012) Transcriptional profile of GTP-mediated differentiation of C2C12 skeletal muscle cells. Purinergic Signal 8: 207–221. 22127439 10.1007/s11302-011-9266-3 PMC3350577

[42] Riquelme MA, Cea LA, Vega JL, Boric MP, Monyer H, et al (2013) The ATP required for potentiation of skeletal muscle contraction is released via pannexin hemichannels. Neuropharmacology 10.1016/j.neuropharm.2013.03.022 23583931

[43] Clark KD, Hennessey TM, Nelson DL, Preston RR (1997) Extracellular GTP causes membrane-potential oscillations through the parallel activation of Mg^2+^ and Na^+^ currents in Paramecium tetraurelia. J Membr Biol 157: 159–167. 9151657 10.1007/s002329900225

[44] Mancinelli L, Fanò G, Ferroni L, Secca T, Dolcini BM (1983) Evidence for an inotropic positive action of cGMP during excitation-contraction coupling in frog sartorius muscle. Can J Physiol Pharmacol 61: 590–594. 6309351 10.1139/y83-090

[45] Uhlen P, Fritz N (2010) Biochemistry of calcium oscillations. Biochem Biophys Res Commun 396: 28–32. 20494106 10.1016/j.bbrc.2010.02.117

[46] Di Capite J, Ng SW, Parekh AB (2009) Decoding of cytoplasmic Ca^2+^ oscillations through the spatial signature drives gene expression. Curr Biol 19: 853–858. 19375314 10.1016/j.cub.2009.03.063

